# Feasibility, acceptability, and effectiveness of school-based dance movement psychotherapy for children with emotional and behavioral difficulties

**DOI:** 10.3389/fpsyg.2022.883334

**Published:** 2022-08-22

**Authors:** Zoe Moula, Joanne Powell, Shirley Brocklehurst, Vicky Karkou

**Affiliations:** ^1^Imperial College London, London, United Kingdom; ^2^Faculty of Health, Social Care & Medicine and Faculty of Psychology, Edge Hill University, Ormskirk, United Kingdom

**Keywords:** dance and movement, psychotherapy, children, schools, wellbeing, quality of life, sleep, randomized controlled study

## Abstract

**Background:**

Schools have been increasingly employing dance movement psychotherapists to support children cope with daily worries and stress, express and understand their emotions, develop self-awareness and self-esteem. However, evidence on the impact of dance movement psychotherapy as a tool for prevention of mental health difficulties in childhood remains limited.

**Methods:**

Sixteen children (aged 7–9) with mild emotional and behavioral difficulties from two primary schools were randomly assigned to a Dance Movement Psychotherapy (DMP) intervention or to a waiting list, within a larger pilot cross-over randomized controlled study which aimed to (a) test whether all elements of study design can work together and run smoothly in a full-scale RCT; and (b) investigate the effectiveness of arts therapies in improving children’s health related quality of life (HRQOL; EQ-5D-Y), wellbeing and life functioning (Child Outcome Rating Scale; CORS), emotional and behavioral difficulties (Strengths and Difficulties Questionnaire; SDQ), and duration of sleep (Fitbits). The therapeutic process was also evaluated through interviews with children, participant observations, the Children’s Session Rating Scale (CSRS), and ratings of adherence to the therapeutic protocol.

**Results:**

The findings indicated that DMP led to improvements in children’s life functioning, wellbeing, duration of sleep, emotional and behavioral difficulties, but not in quality of life. The improvements were maintained at the follow-up stages, up to 6 months post-intervention. Interviews with children also suggested positive outcomes, such as self-expression; emotional regulation; mastery and acceptance of emotions; improved self-confidence and self-esteem; reduced stress; and development of positive relationships. However, children would have preferred smaller groups and longer sessions.

**Conclusion:**

This study indicated that all outcome measures would be suitable for inclusion in a larger randomized controlled trial, though the EQ-5D-Y is not recommended as a stand-alone measure due to its lack of sensitivity and specificity for young participants. The adherence to the therapeutic protocol ratings differed between children and adults, highlighting the need to include children’s voice in future research. Strategies are also proposed of how to conduct randomization of participants in ways that do not hinder the therapeutic process.

## Introduction

### Mental health provision for children in schools

In the United Kingdom, one in five children (equating to 1.1 million) reported feeling unhappy with their lives since the COVID-19 crisis (Children’s Commissioner, 2021). Clinically significant mental health conditions have increased by 50%, while two thirds of primary school children have experienced social isolation and loneliness; also a 50% increase compared to pre-COVID-19 (Children’s Commissioner, 2021). Furthermore, although the number of domestic abuse rose by at least 30%, the number of children coming to the attention of services has fallen dramatically (Children’s Society, 2020). It is therefore not surprising that in a survey of 2,438 children and young people (Young Minds, 2021), approximately 70% said that pandemic will have a long-term negative effect on their mental health.

The World Health Organisation (WHO) (2004) proposes that mental health promotion should be integrated into the school curriculum, reaching children who might not otherwise obtain help. Although school mental health provision varies across countries, the European Union Dataprev project found that over the past 25 years there has been a significant increase in large-scale school mental health programs (Weare and Nind, 2011; Patalay et al., 2017). Having daily contact with children and their families, schools are a key entry point to community-based mental health provision (Stephan et al., 2007; Jané-Llopis and Braddick, 2008; Caan et al., 2014). Their remit as educational institutions helps to reduce stigma, increases inclusivity, while accessing supportive networks of peers, teachers, healthcare professionals, and parents (Kavanagh et al., 2009; Greenberg, 2010). Furthermore, school-based counselors and psychotherapists can streamline the referral process and target children experiencing barriers due to lack of transportation, parent work schedules, funding, and inadequate treatment from other sources (Capp, 2015).

Nevertheless, a major challenge for school mental health services is that the focus relies heavily on the treatment of severe difficulties or disorders, whereas early detection and prevention might be equally important (The World Health Organisation [WHO], 2004; Goldie et al., 2016). When opportunities for prevention are missed, chances are increasing for children to drop out of school, self-harm, become aggressive, violent, or even suicidal. In the United Kingdom, it is estimated that 7,000 children are being excluded annually (equivalent to 35 children/day) while 1,300 of these exclusions come from primary schools (Children’s Commissioner for England, 2017). This might be because more than 70% of children lack supportive services at a sufficiently early age (Children’s Society, 2018), 30% of referrals are turned away and waiting lists can take up to a year (Children’s Commissioner for England, 2017). Such delays in addressing children’s needs have long-lasting and potentially irreversible negative effects, highlighting the importance of support at the early stages of children’s education.

### Dance movement psychotherapy for children in schools

The UK Professional Association for Dance Movement Psychotherapy [ADMPUK] (2013) has defined DMP as *“a relational process in which client/s and therapist engage creatively using body movement and dance to assist integration of emotional, cognitive, physical, social and spiritual aspects of self”* (p. 1). Schools are increasingly employing DMP practitioners to support children cope with stress and worries, express and understand their emotions, develop self-awareness and self-esteem (Karkou, 2010; Moula et al., 2020b; Karkou et al., 2022). Embedding DMP within the educational system, may feasibly, address children’s emerging needs and have a positive impact on their wellbeing, bridging the gap between health and education.

DMP is especially important for children who might not wish, or are unable, to verbalize their thoughts and emotions, especially under stressful circumstances (Moula et al., 2019). This is because DMP emphasizes the externalization of bodily felt emotions in creative ways, aiming to assist children process difficult feelings and integrate new self-soothing strategies (Meekums, 2008). The connection between motion and emotion is integrative in DMP, as movement has an emotional content and the expression of this content forms the basis for the therapeutic process (Berger, 1972; Johnson, 1995). As such, movement serves as an agent of expression of inner tensions and distress and can become a container for old and, as the therapy progresses, of new experiences (Frizzell, 2006). When DMP is delivered to children, some of the key goals include, but are not limited to: understanding and managing difficulties which may interrupt learning; social interaction skills; emotional, physical, and mental integration; self-expression; communication; self-awareness; empathy; self-esteem and self-confidence (Eke and Gent, 2010).

### Evidence-based dance movement psychotherapy for children and young people

Current studies with children and young people showed that DMP can support children with a range of social, emotional, and behavioral difficulties (Goodgame, 2007; Eke and Gent, 2010; Ballard et al., 2014; Moula et al., 2020a). In terms of behavioral difficulties, DMP allows children to develop problem-solving abilities and gain methods of self-control, preventing underlying forms of aggression from being escalated into more disruptive behaviors (Koshland et al., 2004; Koolaee et al., 2014). Preliminary studies have also highlighted the value of DMP for children with ADHD (Grönlund et al., 2005; Redman, 2007), depression (Jeong et al., 2005), and learning difficulties (Lamond, 2010; Mullane and Dunphy, 2017; Unkovich et al., 2017). The impact of DMP is extending to children experiencing stressful events, such as immigration (Bareka et al., 2019; Grasser et al., 2019; Dieterich-Hartwell et al., 2020), pandemics and lockdowns (Muhs, 2021; Shuper-Engelhard and Furlager, 2021). A current systematic review has also highlighted the contribution of DMP for the wellbeing of children with ASD (Aithal et al., 2021). Furthermore, DMP had been used with children and young people in pediatric settings (Zilius, 2010) and psychiatric clinics (Erfer and Ziv, 2006; Anderson et al., 2014) addressing more complex difficulties.

However, what is missing from current evidence is the provision of DMP for prevention and resilience, rather than for treatment purposes for this age group. Existing research and practice in creative psychotherapies is based on children who have been diagnosed with a disorder or disability (Malchiodi, 2012). When existing research addresses prevention, it also tends to focus on older population. A systematic review on school-based creative psychotherapies as a tool of prevention of mental health difficulties (Moula et al., 2019) identified only two eligible DMP studies (Koshland et al., 2004; Abdulazeem, 2014). The former included children with disabilities, while the latter focused on violence prevention. No study looked at DMP with children in mainstream primary education with emotional and behavioral difficulties as a preventive intervention before developing mental health issues. The current study aimed to address this gap, by administering a DMP intervention in children with mild emotional and behavioral difficulties in a school-based setting.

### Aims and objectives

This study was part of a pilot cross-over randomized controlled study delivering arts therapies across primary schools in the Northwest of England, specifically, art therapy, music therapy, dramatherapy, and dance movement psychotherapy. The design of the pilot was grounded on a systematic review (Moula et al., 2020a), which informed the development of the sessions, choice of outcome measures and research methods, while a full account of the study protocol has been published elsewhere (Moula et al., 2019). The primary aim of the pilot was to test whether all components of the study design (e.g., recruitment, randomization, outcome measures, follow-up) can work together and run smoothly in a full-scale RCT. The present article presents a sub-study of this pilot and is focused specifically on the process and outcome evaluation of the DMP intervention. As such, the research questions of this sub-study are:

1. What are the outcomes of DMP as evaluated through:
  a) verbal and non-verbal expressions relating to children’s feelings and thoughts on their wellbeing;
  b) self-reported standardized outcome measures relating to quality of life, wellbeing, and life functioning;
  c) biomarkers relating to children’s duration of sleep?
2. To what extent the DMP intervention adhered to the therapeutic model?

## Methodology

### Methodological positioning

Given the complexity of health difficulties, social interventions require data collection from various perspectives and methods (Johnson et al., 2007; Creswell and Plano Clark, 2017). In this study, mixed-methods were used to evaluate the DMP process and outcomes from children’s experiences and standardized measures. This approach is philosophically underpinned by pragmatism (Howe, 1988; Creswell, 2014). Pragmatism embraces both the positivist/postpositivist and constructive paradigms aiming to gain a comprehensive understanding of the research problem both from quantitative (measured facts) and qualitative methods (personal experiences) (Onwuegbuzie and Johnson, 2006; Brierley, 2017). Epistemologically, there are times during the research design that an objective stance is adopted using standardized measures, while at other times a subjective approach is followed through attempting to understand participants’ perceived realities (Teddlie and Tashakkori, 2009) and thus, producing socially useful knowledge (Yvonne Feilzer, 2010).

### Participants

Sixteen children who experienced mild emotional and behavioral difficulties were recruited from two primary schools across the Northwest of England. The eligibility criteria for participation are presented in Table 1. Half of the children were assigned to the DMP intervention group immediately after randomization, while the other half acted as control group in the beginning and received the DMP intervention 3 months later (waiting list). Detailed information about the randomization method and sample size calculations are provided in the study protocol (Moula et al., 2019). The number of children who were recruited for the DMP intervention was small because this was a sub-study of a larger pilot study (Moula et al., 2020b).

**TABLE 1:** Eligibility criteria.

**Inclusion criteria:**
• Children aged 5–12 years’ old • Children who experience mild emotional and behavioral difficulties based on teachers’ ratings in the Strengths and Difficulties Questionnaire with impact supplement for the teachers of 4–17 years’ old • Children with a diagnosis of disabilities that are not expected to affect them in participating in group arts therapies, including, but not limited to, dyslexia, dysgraphia, dyscalculia, intellectual disabilities • Children who are not already involved in arts therapies, other forms of psychotherapy or mental health support • Children that both themselves and their parents or legal guardians consent to their participation in the study • Children with or without fluency in English
**Exclusion criteria:**
• Children younger than 5 or older than 12 years’ old • Children whose scores from teacher rating on the Strengths and Difficulties Questionnaire are higher than mild emotional and behavioral difficulties • Children with a diagnosis of disabilities that are expected to need one-to-one support, including, but not limited to, autistic spectrum disorder, attention deficit hyperactivity disorder, cerebral palsy, Down syndrome, epilepsy, cystic fibrosis, spina bifida, multiple sclerosis, depression, anxiety disorder, hearing impairment, visual impairment. This exclusion was made on the basis that the arts therapists might have not been able to meet children’s needs within a large group of 6–8 children • Children who require one-to-one support for any other reason • Children who attend extra-curriculum activities at the same time as the arts therapies and thus are likely to miss arts therapies sessions • Children who already participate in arts therapies or other forms of psychotherapeutic input • Children who consent to participate, but their parents or legal guardians do not consent; and vice versa

For the first cohort, four girls and four boys (aged 8–9) were selected through a random number generator software. However, two boys withdrew their participation before the beginning of the sessions on the basis that “dance is not for boys.” Following their withdrawal, the other two boys expressed that they would feel uncomfortable and embarrassed if they were surrounded by girls and they decided to withdraw as well. Following discussions with the class teachers, we decided to recruit only girls for the first cohort of DMP. For the second cohort, four girls and four boys were recruited (aged 7–8). However, we emphasized that the sessions would be focused on movement, rather than on dance, and there were no withdrawals. As such, the final sample who participated in DMP consisted of 12 girls and four boys.

### Dance movement psychotherapy intervention

**FIGURE 1:**
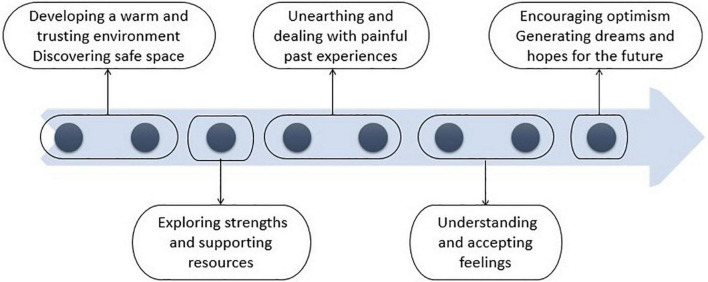
Therapeutic model.

DMP was delivered 1 h weekly, across eight consecutive weeks. Each DMP session was focused on a specific topic and therapeutic goal (Figure 1 and Table 2; see also Moula et al., 2019; Moula, 2020), based on existing evidence of previous interventions in arts therapies (Moula et al., 2020a), the Arts for the Blues model developed for adults with depression (Omylinska-Thurston et al., 2020) and the wider literature in school-based arts therapies (Karkou, 2010). It also drew on DMP theory and practice and the work of Marian Chace in particular (Chaiklin and Schmais, 1986).

**TABLE 2:** Session structure and activities.

1. **Opening circle:** In the early sessions, the opening circle included simple activities, such as name games. In later sessions, children were asked to indicate how they were feeling through a movement, gesture or facial expression.
2. **Warm up:** In the first session, children were taught the sign language for the Confidentiality Rap, which then turned into a dance. This became a regular and popular part of the warm-up part for subsequent sessions. Most sessions included a Chacian circle whereby children would connect to their body by marching, tapping, stretching and circling body parts, moving into group improvisations and followed by free dance. During the warm-up, deep pressure self-touch was used to establish body boundaries; exploring self-space and general space, grounding movements, and contrasting movements, giving children time to prepare for the semi-structured part of the session.
3. **Semi-structured activities responding to the main session goals:** Activities were designed specifically for each week’s theme. This part often started by inviting children to close their eyes and reflect on the week’s theme (e.g., “safe space”). They were then invited to show with movements, actions, or expressions, how they would represent those aspects of themselves or their lives. For example, using buddy bands they explored how to stay safe and look after each other. To facilitate emotional expression, children were involved in activities such as body mapping or using ropes to outline their body shapes on the floor, where children reflected on their emotions and placed props on the body map to show where they were felt these emotions in their body. In some sessions it was necessary to work on building trust before moving to this part of the sessions. Example activities included: (a) joining hands - leaning back/forward, taking one leg off the ground; (b) pressing palms—giving and receiving weight or pressure, allowing weight to shift; or (c) group circle using the buddy band, pulling and pushing, and trusting that we will keep each other safe.
4. **Children’s artistic summary:** Closer to the end, each session involved an artistic summary, such as creating a group sculpt, or using a movement from each child to make a group choreography. In the last session, children created a timeline. In this activity, each child laid a length of rope on the floor, representing the timeline from the first session to the last. The group reflected together on each session, and the children placed an object on the line to represent how they felt, what they learned and what they would take forward. They were also given the opportunity to create and keep a diary reflecting on their individual work, working as a group, and what they would take with them after the end of the sessions.
5. **Closing:** To end the sessions, children were involved in activities such as rocking, traveling around the room, shaking hands, making eye contact, acknowledging one another, and finally guided relaxation and grounding.

The DMP (SB) was trained on the protocol application and was encouraged to make her own clinical judgments moderating the session structure when needed. The DMP has been teaching dance and movement for over 45 years. She was trained and qualified as DMP 10 years ago and working as DMP since then. The protocol was used to evaluate whether the sessions adhered to the therapeutic model, and modifications were recorded for fidelity.

### Data collection and analysis

**FIGURE 2:**
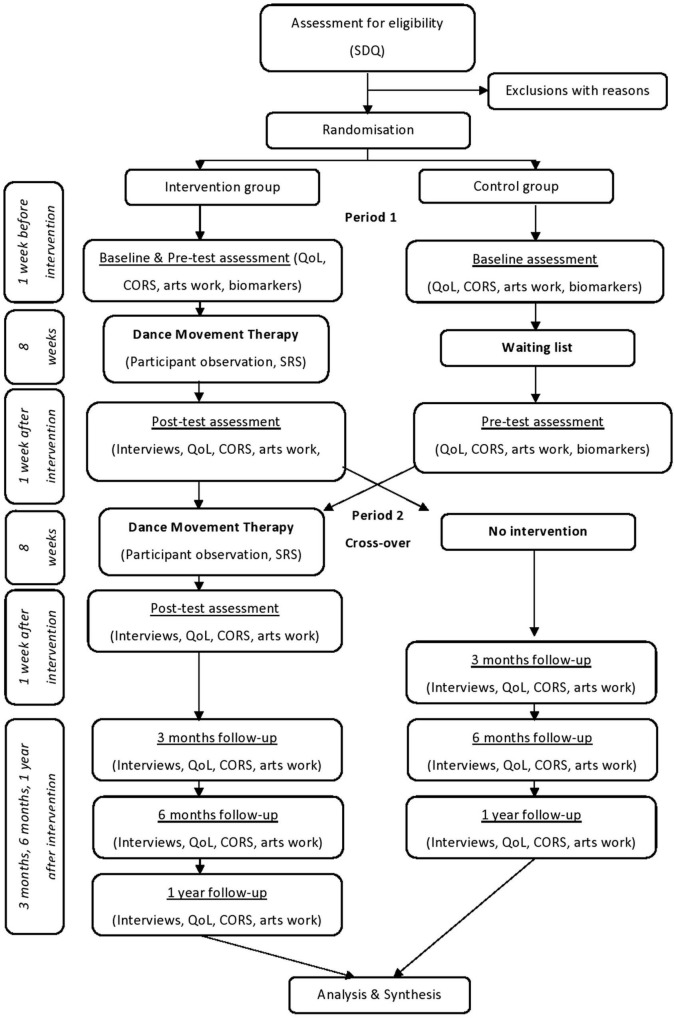
Procedure flowchart.

The outcome evaluation was completed by interviews, questionnaires, and biomarkers (FitBits). The process evaluation was completed by participant observations, ratings of adherence to the therapeutic protocol, and children’s session ratings. A flowchart of the procedure is presented in Figure 2.

### Interviews

Semi-structured one-to-one interviews were conducted at the end of the intervention to understand children’s perspectives regarding what they found helpful or unhelpful and what they enjoyed or did not enjoy. To facilitate memory retrieval, 1-2 photographs from each session were selected and showed to the children. The interviews were analyzed through reflexive thematic analysis (Braun and Clarke, 2006). To minimize power imbalance and avoid misinterpretations of children’s views, member cross-checking was employed (Brooks et al., 2017). All tentative interpretations were made available to children to express whether they represented their own viewpoints and to evaluate their accuracy (Atkinson et al., 2003). The supervisory team (VK, JP) also reviewed the interpretations and provided further insights. Both methods contributed to credibility improvements (Higgins and Green, 2011).

### Adherence to therapeutic protocol

The lead researcher (ZM) was a participant observer in all sessions. At the end of each session, the researcher (ZM) and DMP (SB) rated the adherence to the therapeutic protocol and recorded any changes for fidelity purposes. Participant observation was supported by video recordings, allowing us to capture simultaneous complex interactions and observe events retrospectively (Asan and Montague, 2014). A rating was given for each of the 10 therapeutic principles by the therapist (Rater A) and the researcher (Rater B). The agreement between raters was assessed using the Bland-Altman plot system (Bland and Altman, 1986). This allowed the identification of systematic differences between the raters, which were classed as “fixed bias” or outliers. The mean score was calculated (A + B/2) along with the difference between rater scores (A - B). The overall degree of adherence from both raters estimated the inter-rater reliability. By exploring the level of intersubjectivity, this method improved the dependability of the findings (Higgins and Green, 2011).

### Biomarkers

Biomarkers (FitBits) were used to capture changes in children’s duration of sleep. Children wore the Fitbits for 3 days, 1 week before and 1 week after DMP. The number of minutes asleep was calculated as the total number of minutes asleep minus the number of active minutes during the night. This calculation provided an overall measure of sleep duration, recorded in minutes. Duration of sleep was averaged across the 3 days pre-intervention and 3 days post-intervention.

### Questionnaires

All children were administered a standardized battery of tests, and one questionnaire was administered to teachers. The child-reported questionnaires were the: Quality of Life for Children (EQ-5D-Y) (Wille et al., 2010); Child Outcome Rating Scale (CORS) (Low et al., 2012); Child Session Rating Scale (CSRS) (Low et al., 2012). The teacher-reported questionnaire was the Strengths and Difficulties Questionnaire with impact supplement (SDQ) (Goodman, 2001). A summary of all measures is presented below, while the study protocol (Moula et al., 2019) provides a more detailed description.

The *Quality-of-Life scale for Children* (EQ-5D-Y) (Wille et al., 2010) reflects young people’s own judgments of their health-related quality-of-life. It includes dimensions of activity level, self-care, doing usual activities, experiencing pain or discomfort, and feeling worried, sad, or unhappy. Test–retest reliability ranges between 69.8 and 99.7% (Ravens-Sieberer et al., 2010). Kappa coefficients reach up to 0.67, while the correlation coefficients with other measures of self-rated health indicate convergent validity up to *r* = –0.56 (Ravens-Sieberer et al., 2010).

The *Child Outcome Rating Scale* (CORS) (Low et al., 2012) evaluates areas of life functioning and wellbeing that might change following a therapeutic intervention. It includes individual wellbeing, interpersonal wellbeing, social wellbeing, and overall wellbeing. Research found moderate to high reliability (Bringhurst et al., 2006), moderate test-retest reliability (Bringhurst et al., 2006), and moderately strong concurrent validity with longer, more established measures of treatment outcome and therapeutic alliance (Miller et al., 2003; Bringhurst et al., 2006).

The Child Session Rating Scale (CSRS) (Low et al., 2012) assesses key dimensions of effective therapeutic relationships and children’s feedback on therapeutic progress. The CSRS was administered at the end of each session to get real time feedback from children so that any alliance difficulties could be identified and addressed (Miller et al., 2003). Existing research has demonstrated good reliability, test re-test reliability, and concurrent validity (Miller et al., 2003; Campbell and Hemsley, 2009).

The *Strengths and Difficulties Questionnaire* (SDQ) (Goodman, 2001) with impact supplement for teachers is an emotional and behavioral screening tool. The impact supplement examines the nature of difficulties, such as burden to others, social impairment, chronicity and distress. Existing evidence suggests strong internal consistency (Yao et al., 2009); moderate test-retest reliability (Yao et al., 2009); good concurrent validity (Muris et al., 2003); and good discriminant validity (Lundh et al., 2008).

IBM’s SPSS (version 25) was used for calculating means and standard deviations in the above questionnaires. Descriptives, including means and standard deviations, for all the variables in the study are presented and discussed within the results. Descriptives for the CSRS across the eight sessions as well as CORS and HRQOL at follow-up points (3- and 6 months post-intervention) are also presented.

## Results

### Context of dance movement psychotherapy provision in two schools

In the first school, eight children were selected from two classes (aged 8–9). However, two boys decided that “dance is not for boys” and withdrew. Following this, the other two boys expressed that they would feel uncomfortable being surrounded by girls, and they also withdrew. We therefore recruited eight girls, as other boys would feel embarrassed to participate at this stage. In this first school, there was a need for a safe and private environment that was lacking. There were frequent interruptions while the safeguarding officer required that a permanent member of staff should observe every session. However, the presence of an external non-participating observer impacted on the group who hesitated to share experiences, feelings, or thoughts in front of them. Following the first cohort’s completion, we decided to terminate the collaboration with this school.

The second cohort started in a new school, where a safe and private environment was secured this time. Eight children from two classes were recruited (aged 8–10). The study was presented as “movement therapy” to avoid dropouts from boys; there were no dropouts. Although privacy and safety were ensured, the hall was particularly spacious, requiring extra effort to contain the group. In addition, some children required attention regarding their listening skills, which created challenges for the therapeutic process. As a result, the therapist needed to modify the intervention to meet these needs as presented in the ratings of adherence to the therapeutic protocol below.

### Qualitative findings

The interviews revealed that the activities that children enjoyed the most were: (a) experimenting with different fabrics and materials (e.g., scarfs, parachutes); (b) stories and role-playing; (c) mindfulness techniques (e.g., deep breathing); (d) exploring their safe space; and (e) identifying their strengths. These activities offered to children space to work individually as well as being part of the group. They also encouraged children to treat each other with kindness and respect the process of emotional sharing within a safe and non-judgmental environment. For example, one child said that:

*“It was really good to share what we felt through our movements, you know, that we could share anything we feel [*…*] this was our safe space”*

Particularly the “Confidentiality Rap,” which was the first song of every session, was children’s favorite song because it reminded them that anything that was shared in the group was confidential. For example:

*“I loved the song [Confidentiality Rap], my favourite part ‘what we say in the group, stays in the group, it’s confidential [*…*] it was just to make sure that we are all protected”*

The aspects of DMP that children found most helpful were being part of a team, looking after each other, and knowing that there was someone to speak to when they needed support. As a child said:

*“I was helping others to feel better, some people can get upset very easily and it helped me to understand that sometimes they don’t mean it and they feel sorry. I think it helped us to become better friends”*

Furthermore, children mentioned that DMP had a calming effect on them, especially when they experienced frustration and/or when they were dealing with uncomfortable situations:

*“When I felt upset it helped me calm down [*…*]. I’m fighting less with my brother now and I can control my anger better”*

*“When I am frustrated, I know that there are better ways to do it than being mad at other people when they haven’t really done anything and it’s not their fault”*

Although there were no activities that children found unhelpful or did not enjoy, there were some aspects of the sessions that most children did not enjoy, specifically the arguments and the lack of listening. Even sensing lack of listening from one member was enough to interfere with the therapeutic process and prevent others from sharing. As a child said:

*“I didn’t like shouting at each other, I would prefer smaller groups maybe like five because when we argue we lose time and it’s not fun”*

As discussed below and as children recommended, working with smaller groups could improve children’s experience and better facilitate the therapeutic process.

### Quantitative findings

#### Child outcome rating scale and health related quality of life

Table 3 shows the means and standard deviations for CORS and HRQOL post-intervention and at follow-up (i.e., 3-, and 6-months post-intervention) for those that took part in the intervention (*N* = 16) and from the control intervention (*N* = 8) as well as the total sample (*N* = 24).

**TABLE 3:** Means and standard deviations (bracketed) for CORS and HRQOL post-intervention and at follow-up at 3-months and 6-months post-intervention, for total sample and separated by intervention group.

	Controls (*N* = 8)	Intervention (*N* = 16)	Total (*N* = 24)
CORS post-intervention	27.63 (10.77)	33.47 (5.79)	30.96 (8.30)
CORS 3-months	–	30.47 (7.29)	–
CORS 6-months	–	34.03 (7.87)	–
HRQOL post-intervention	29.64 (13.75)	30.79 (9.01)	30.17 (10.42)
HRQOL 3-months	–	33.19 (10.45)	–
HRQOL 6-months	–	34.73 (12.93)	–

The DMP intervention group presented higher post-intervention scores than the control group for both CORS (i.e., 33.47 ± 5.79 vs. 27.63 ± 10.77) and HRQOL (i.e., 30.79 ± 9.01 vs. 29.64 ± 13.75). For those that took part in the DMP intervention mean scores for CORS showed lowest scores pre-intervention (mean = 25.93 ± 8.42), which increased post-intervention (mean = 33.47 ± 5.79; mean difference = 7.54) and further still at 6-months follow-up (mean = 34.03 ± 7.87), though this difference was fairly small (i.e., mean difference = 0.56) and was preceded by a drop in score at 3-months. In contrast HRQOL followed a continuous upward trajectory from post-intervention to 3-months and then 6-months follow-up for those that took part in the DMP intervention (see Table 3 for mean scores).

#### Strengths and difficulties questionnaire and sleep duration

Mean SDQ score was higher post-intervention (mean = 9.19 ± 5.49) than pre-intervention (mean = 7.88 ± 4.50) with a mean difference of 1.313 (*SD* = 3.04, 95%CI: –2.94, 0.31); this represents an improvement on this measure relating to teachers’ assessment of the child. Descriptive statistics indicate a higher mean number of minutes sleep post-intervention (mean = 485.00 ± 48.65) than pre-intervention (mean = 466.58 ± 59.59) with a mean difference of 18.42 min (*SD* = 43.68, 95%CI: –46.17, 9.34). This indicates that children slept approximately 18 min longer post-intervention than pre-intervention.

#### Child session rating scale

**FIGURE 3:**
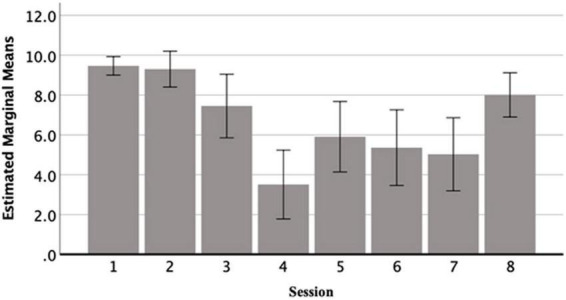
CSRS mean scores for each DMP session.

Figure 3 shows a bar chart presenting mean scores and error bars ratings across the eight DMP sessions. The figure shows that session quality was rated highly to begin with, specifically a mean of 8.47 (±0.73) was given for session 1, while sessions 2 and 3 received a mean score of 9.30 (±1.42) and 7.45 (±2.51) respectively. However, session quality dipped in the middle with a low of 3.51 (±2.72) for session 4 and ratings of 5.91 (±2.78), 5.36 (±2.99) and 5.03 (±2.89) for sessions 5, 6, and 7 respectively. However, session quality ratings increased in the last session with a mean score of 8.01 (±1.75), which is close to the session 1 ratings.

#### Adherence to therapeutic protocol

**FIGURE 4:**
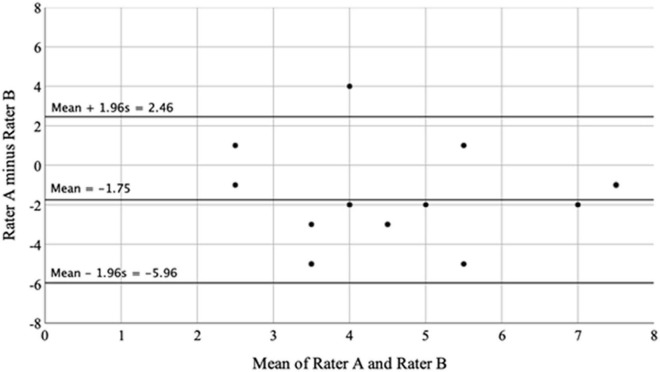
Bland-altman plot showing the difference between raters plotted against the mean of the two raters.

A rating was given for each of the 10 conditions by each rater for each group, yielding a total of 20 scores for the therapist (Rater A) and the researcher (Rater B). The Bland-Altman plot (Bland and Altman, 1986) evaluated the raters’ agreement. The mean score for raters A and B, for each of the 20 measurement points was calculated (A + B/2) along with the difference between rater scores (A - B). An overall disagreement of ±2 units or less was considered to be within the acceptable limits of agreement. The average of differences (*d̄*) between raters was –1.75, which was considered to be acceptable. This indicates that, on average, rater B (researcher) gave a rating of 1.75 units higher than rater A (therapist). The Bland-Altman plot (Figure 4) represents the difference between raters (A - B) against the mean of the two raters. The bias was –1.75 units, which is represented by the gap between the x-axis at 0 corresponding to zero differences, and the parallel line to the x-axis at –1.75 units.

There did not appear to be any systematic bias, with no clear concentrations of data within, above or below this gap. The lack of agreement was summarized by calculating the bias estimated by the mean differences (*d̄*) and the standard deviation. We expected most of the differences to lie within *d̄*-1.96*s* and *d̄* + 1.96*s* if the differences were normally distributed (Gaussian). In this study, s = 2.15, so that 95% of differences were within –5.96 and 2.46. These figures represented the lower and upper limits of agreement respectively. This showed that ratings made by rater A may be –5.96 units below or 2.46 units above rater B. It is important to note that the Bland-Altman plot did not indicate whether the agreement was sufficient or suitable to use one rater or the other indifferently. It also did not quantify the bias and the range of agreement within which 95% of the differences between one measurement and the other were included (Giavarina, 2015).

A one-sample *t*-test was used to determine whether the mean of difference between raters differed significantly from 0, with 0 representing no difference. A significant result indicated the presence of a fixed bias. Results from the one-sample *t*-test were significant (*t*_19_ = –3.642, *P* = 0.002, mean difference = –1.75, 95% CI: –2.76, –0.74). This indicated that rater A (therapist) was systematically rating less the adherence than rater B (researcher), with an average of 1.75 units less than rater B. This suggested that, if the therapist was considered the ideal candidate (or “gold standard”) in determining adherence to the therapeutic protocol, the researcher would not be an adequate substitute.

## Discussion

Overall, the study design was followed as planned, however, some challenges which were encountered need to be considered in future studies. The main recruitment challenge in the DMP intervention was the term “dance.” Boys were reluctant to participate because of gender stereotypes and negative associations between men and dance. Replacing the term DMP with “movement therapy” made the recruitment easier. Another challenge was the provision of a safe therapeutic environment. The rooms were exposed to loud noises and interruptions, a challenge commonly faced in school-based psychotherapies (Danieli et al., 2019). The presence of an external observer, which was required from the first school for safeguarding purposes, interfered with children’s sense of safety and made children reluctant to share openly. It became necessary to seek an alternative school for the purposes of allowing for the DMP intervention to take place according to the protocol.

During the interviews post-intervention, children expressed improvements in their sense of wellbeing, creativity, and resourcefulness. They also discovered the multisensory potential of objects, such as in parachutes, fabrics, scarfs and other soft materials. This links back to the literature regarding creativity and imagination as one of the essential therapeutic elements (Karkou and Sanderson, 2006) that enables: access to difficult unconscious materials (Case and Dalley, 1992); safe self-expression (Lee, 2015); pleasure from artistic engagement (Csikszentmihalyi, 2002); and increased sense of empowerment (Zimmerman, 2000).

Children expressed that they felt more “confident” and “special.” Some children noted that they learned who is their “true self” and they felt less afraid to show this “true self” to others. Therefore, empowerment came alongside self-acceptance; both of which are key therapeutic ingredients (Sutherland et al., 2010; Sassen, 2012). Though self-acceptance, the acceptance of others also grew as children mentioned that they were trying more to understand others, rather than judging or blaming them. This might indicate that children developed ownership over their own behaviors and actions, and increased sense of self-control; both of which are key elements of the empowerment theory (Zimmerman, 2000).

Overall, the structure of the sessions remained consistent to the therapeutic protocol and no major modifications were recorded. Children’s ratings of session quality were higher for those sessions they said included enjoyable and entertaining activities. Children also rated higher the sessions with highly structured activities, indicating that they might prefer sessions which are well-structured and to some extent directive. These findings underline the value of triangulating the sources of information and the need to include children’s voice in research.

There were significant differences in the adherence ratings between the researcher (ZM) and the DMP (SB). It is possible that the DMP practitioner was more self-critical and reluctant to rate her own sessions too high. Vice versa, the researcher was more likely to rate the sessions higher due to confirmation bias: the researcher’s tendency to look for information or patterns in data that confirm their hypotheses, consciously or unconsciously. Nevertheless, the ratings helped to understand which therapeutic principles were achieved or were more difficult to be achieved. Finding balance between verbal and non-verbal communication was the most challenging therapeutic principle. This was because some children were very talkative, allowing less or no space for non-verbal communication, while other children were quiet, shy, and had limited verbal interactions with the group. Revisiting past experiences with direct impact on the here and now was also challenging for the sessions where conflicts and tension arose. It is likely that the therapeutic environment lacked the appropriate safety to openly revisit past experiences and it highlights the importance of establishing safety prior to opening up and sharing with the group. Working with empathy and warmth was the only principle that was met in all sessions.

### Outcome measures: Child outcome rating scale, health related quality of life, strengths and difficulties questionnaire, and sleep duration

DMP had a positive effect on CORS, which were higher post-intervention for those that took part in the DMP intervention than the control group. The same was not true for HRQOL where post-intervention HRQOL scores were very similar between intervention and control groups. Previous research has found that DMP can significantly improve the quality of life of children and young people (i.e., Karkou et al., 2010; Madden et al., 2010; Abdulah and Abdulla, 2018). Moderate effects have been also found in DMP systematic reviews (Bradt et al., 2015), and meta-analyses (Koch et al., 2014, 2019). However, these reviews included children as well as young people and adults. Since there is no systematic review looking at the effectiveness of DMP for children’s quality of life, the existing evidence remains inconclusive.

According to the teachers’ questionnaires (SDQ), children presented fewer emotional and behavioral difficulties following the DMP intervention. These findings echo the results from previous research (Karkou et al., 2010; Abdulazeem, 2014; Alotaibi, 2017), which concluded that teachers noticed significant reduction in children’s emotional, conduct, hyperactivity, and peer relationships difficulties, as well as improvements in prosocial behavior.

Improvements in duration of sleep was also found through biomarkers (FitBits) with children sleeping approximately 18 min longer post-intervention than pre-intervention in the DMP group. These results were important considering the impact of sleep health on physical and psychological wellbeing. Particularly, sleep is a major influential factor of neurologic function that helps to maintain wellness, increase resilience (Raven et al., 2018), and even change perceptions of pain (Mondanaro et al., 2017). This is because neurologically, the mind remains active during sleep, unconsciously processing thoughts that have been put aside or dismissed (Feld and Born, 2017). Current evidence suggests that creative psychotherapies can soothe and sedate (Loewy, 2020), increasing comfort and decreasing anxiety impeding sleep capacity (Lund et al., 2020). Although this is the first study to explore changes in children’s sleep, preliminary evidence shows that creative psychotherapies improve sleep quality in adults (Wang et al., 2014; Kavurmaci et al., 2019) as well as in infants and toddlers (Loewy, 2020); while evidence comes primarily from music therapy.

This study found that both HRQOL and CORS improvements remained reasonably stable at the 6-month follow-up, indicating that DMP had a long-lasting impact for up to 6 months post-intervention. The systematic review that informed this pilot (Moula et al., 2020a) showed that only one out of the six included studies explored the long-term outcomes of school-based creative psychotherapies. The lack of follow-up or short follow-up may underestimate the benefits and fail to detect hazards, both of which can take longer to emerge (Llewellyn-Bennett et al., 2016). Therefore, long-term follow-up is highly recommended in future research to ascertain the strength of DMP over an extended period and the long-term impact of change on children’s life.

## Limitations and future directions

Due to randomization, some children who did not get along with each other, were placed in the same group. Difficult dynamics in the group resulted in them hesitating to share personal experiences and feelings. If this study was to be replicated in the future, alternative methods of randomization would be recommended, such as cluster randomization. This could reduce the risk of harm that comes with mixing children who do not get along with each other and would enable the group to have more productive intervention. This could also result in closer adherence to the therapeutic protocol, making the intervention more replicable in future studies. Meeting each child separately before their allocation into groups would be highly recommended to understand whether they need one-to-one or group support and whether they can handle sharing the therapist’s attention. Offering a workshop to the school staff at the beginning would also be beneficial to secure understanding of the study, the intervention, and appropriate referrals.

The above suggestions, however, have direct implications on the cost of the study and, long term, the intervention itself. If this strategy was to be implemented, its cost-effectiveness should also be considered. Future studies that calculate the cost-effectiveness of the intervention are urgently needed to establish whether interventions such as this can offer emotional support to children in ways in which not only add value to the school environment but also offer a cost-effective solution to the development of long-term and/or more serious mental health problems.

It is suggested that future research should explore randomization strategies to overcome factors that hinder the therapeutic process, such as mixing children who do not get along well with each other. Cluster randomization or meeting each child one their own prior to allocation into groups could be beneficial strategies in future large-scale studies. If these strategies are employed, cost-effectiveness assessment should also be incorporated.

As this was a sub-study of a pilot, the aim was not to provide evidence that the proposed intervention is demonstrating a statistically significant improvement in our outcome measures, rather it is to test the study design (e.g., recruitment, randomization, follow-up) in terms of its implementation and chosen outcome measures to inform a future large-scale RCT design. Therefore, the focus is on presenting only descriptive statistics from the key outcome measures.

However, since this intervention was delivered to groups of children, rather than individual children, efforts to scale-up this or similar interventions should account for the methodological issues stemming from this study design, known also as Individually Randomized Group Treatment Trial (Pals et al., 2008). More specifically, it is important to consider that the participants in these groups share a similar history (e.g., attending the same school, in the same geographical location), share the treatment environment and interact with each other. As such, intraclass correlation can develop over time in group interventions, violating the major assumption of independence underlying the principal statistical methods used in RCTs (Pals et al., 2008). For this reason, expected intraclass correlations (ICCs) should be taken into account in future sample size estimations. This is expected to increase the required sample size given that the between-group heterogeneity and degrees of freedom should be based on the number of groups rather than the number of group members (Hoover, 2002). However, this method would ensure that the significance of the findings is not overestimated.

Furthermore, as the DMP intervention lasted approximately three months and the researcher (ZM) co-facilitated all sessions, the relationship between the children and the researcher evolved over time. It is therefore possible that children gave higher ratings in the questionnaires because of the close relationship with the researcher. The researcher’s dual role should be taken into account in the appraisal of the findings and in future similar interventions.

As this is a sub-study of a larger pilot randomized controlled study, our findings are limited to sixteen children from four classes and two schools within similar geographical locations. Considering the highly contextualized nature of childhood, the impact on children within different contexts may be different. Therefore, the findings need to be considered in combination with previous studies, such as those summarized in the literature review and discussion section.

## Conclusion

The current study highlights the importance of using a mixed-methods approach to better understand the utility of creative arts-based interventions targeting mental health and wellbeing in mainstream schools. Utilizing both qualitative reports and quantitative measures the current study was able to demonstrate the viability of the DMP protocol and sensitivity of key outcome measures in a school-based setting. Moreover, the current study shows promising findings on the use of a DMP intervention in children in mainstream schools. Improvements were found for children’s wellbeing, life functioning, and sleep duration, as well as emotional and behavioral difficulties assessed by their teachers. Improvements in measures of wellbeing and quality of life were maintained at follow-up, until 6-months post-intervention. Although quality of life has been used extensively in public health research, it is not recommended to be used as a stand-alone measure due to its lack of sensitivity and specificity. In future interventions, widening the targeted outcomes is recommended to capture the impact more precisely. Within the qualitative reports, children expressed that they experienced positive outcomes, such as: self-expression; emotional regulation; mastery and acceptance of emotions; improved self-confidence, self-esteem, and self-worth; reduced stress; and development of positive relationships. Within the session ratings, children rated higher the sessions with entertaining activities and structured activities, indicating that they might prefer sessions which are well-structured and, to some extent, directive. These findings underline the value of collecting information from various sources, including children’s voice in research, while the involvement of children in the development of future outcome measures is also highly recommended. Overall, the study showed the effectiveness of the DMP intervention on children’s wellbeing and duration of sleep, however, several limitations to the study design are highlighted along with proposals for future studies, in particular with regards to randomization, statistical analysis and inclusion of outcome measures.

## Data availability statement

The original contributions presented in this study are included in the article/Supplementary material, further inquiries can be directed to the corresponding authors.

## Ethics statement

The studies involving human participants were reviewed and approved by Edge Hill University, Faculty of Health, Social Care, and Medicine. Written informed consent to participate in this study was provided by the participants’ legal guardian/next of kin.

## Author contributions

ZM: conceptualization, methodology, investigation, analysis, writing—original draft, and visualization. JP: conceptualization, methodology, analysis, writing—review and editing, supervision, and visualization. SB: design and delivery of intervention and writing—review and editing. VK: conceptualization, methodology, writing—review and editing, and supervision. All authors contributed to the article and approved the submitted version.
